# Approach to Macrodactyly: A Case Report and Diagnostic Algorithm for Syndromic and Isolated Forms

**DOI:** 10.3390/pediatric17020032

**Published:** 2025-03-07

**Authors:** Ioannis Kyriakidis, Iordanis Pelagiadis, Nikolaos Katzilakis, Eftichia Stiakaki

**Affiliations:** Department of Pediatric Hematology-Oncology & Autologous Hematopoietic Stem Cell Transplantation Unit, University Hospital of Heraklion & Laboratory of Blood Diseases and Childhood Cancer Biology, School of Medicine, University of Crete, 71003 Heraklion, Greece

**Keywords:** macrodactyly, congenital deformities, megalodactyly, PI3K, overgrowth syndrome

## Abstract

Background: Macrodactyly (megalodactyly or digital gigantism) is a rare condition of overgrowth affecting one or more fingers or toes. Methods: We report a case of a 16-year-old Caucasian male with macrodactyly, lipomas, nevi, dysmorphic features, and autism. The clinical suspicion for a Proteus-like syndrome was high. Results: Targeted *PIK3CA*, *AKT1*, and *PTEN* sequencing for the affected tissue was negative. Subsequent genetic testing revealed a 16p11.2 duplication along with a heterozygous pathogenic variant in *PRRT2* (not causally associated with digit malformation). Conclusions: The clinical management of syndromic macrodactyly is well described by consensus guidelines, but isolated macrodactyly also needs pediatricians’ attention and warrants a multidisciplinary approach. After reviewing the literature, a diagnostic algorithm for the approach and differential diagnosis of macrodactyly is provided. Phenotypes associated with PI3K/AKT/mTOR pathway mutations (including PIK3CA-related overgrowth spectrum PROS) are described. Late effects, follow-up schedules, and surveillance for cancer are discussed.

## 1. Introduction

Macrodactyly (also known as megalodactyly or digital gigantism) is a rare condition characterized by overgrowth affecting one or more fingers or toes, which is usually congenital, benign, and non-hereditary. The incidence of macrodactyly in newborns is approximately 1 in 100,000, accounting for 0.9% of upper-extremity congenital anomalies [1]. In macrodactyly, the fingers or toes enlarge disproportionately, often asymmetrically, causing stiffness, limited mobility, discomfort, or pain, with variable proportions of bone and fat tissue overgrowth unique to each patient [2]. This entity typically follows the growth deregulation of one or more underlying tissues (osseous, nervous, lymphatic, vascular, fibrofatty) or the abnormal accumulation of substances in one or more digits. Flatt’s classification with Upton’s modification is the most commonly used guideline [3]. It may occur as an isolated deformity or as a part of a syndrome. Besides cosmetic concerns, surgical treatment is inevitable in patients who experience functional impairment, even though the rate of recurrence is high [4].

This study aims to present the case of an adolescent with Proteus-like syndrome and rare cytogenetic findings and introduce a diagnostic algorithm from the pediatrician’s perspective.

## 2. Methods

**Figure 1:**
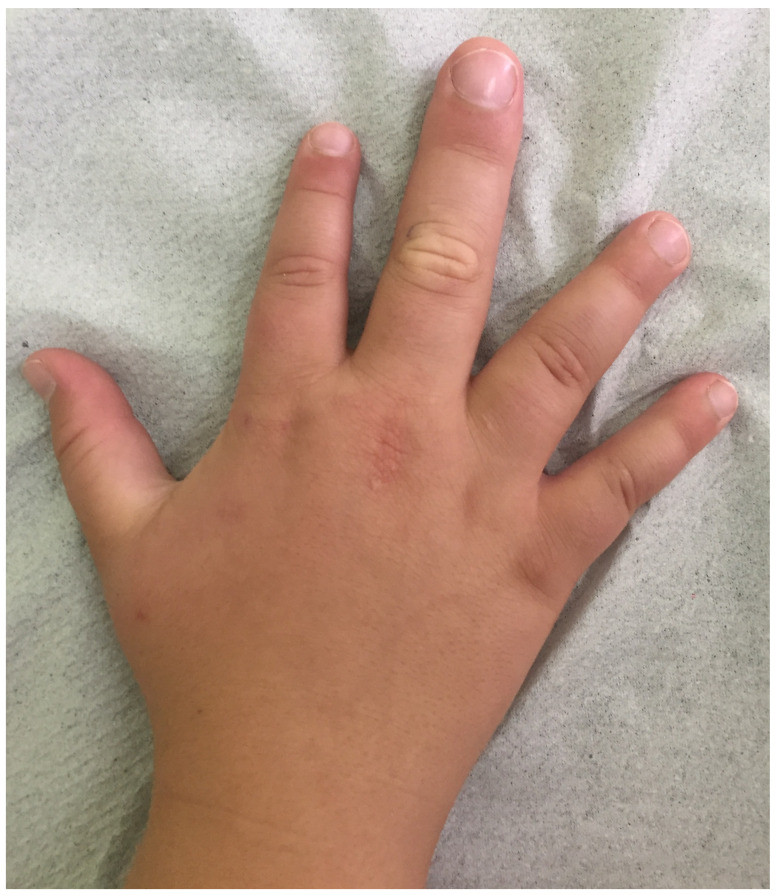
Macrodactyly in our patient.

A 16-year-old Caucasian male was admitted with macrodactyly (Figure 1) and associated functional disorders, along with dysmorphic features and autism spectrum disorder (ASD). Informed consent was obtained from his parents. Following the Declaration of Helsinki and adhering to CARE guidelines (https://www.equator-network.org/reporting-guidelines/care/; accessed on 17 February 2025), we report that his prenatal history was unremarkable. However, his mother has bipolar disorder, migraines, and microcephaly (otherwise phenotypically normal) and gave birth to him at age 40. He was delivered full-term, with a normal birth weight and no early health concerns reported. Clinical evaluation revealed macrodactyly of the middle finger on his right hand, multiple epidermal nevi, and three lipomas on his trunk, as well as kyphoscoliosis, wide nostrils, an open mouth, and obesity (weight and body mass index at the 99th percentile for age and sex). Investigations for his heart murmur returned negative results for cardiac pathology. Regarding psychomotor development, the adolescent was found to have a low average intelligence quotient (86), with a history of slight delays in walking, significantly delayed speech, and one additional year spent in elementary school. Despite receiving special therapies for over ten years, he still exhibits some abnormal fixations. He can now talk and read simple texts or maps well but struggles with writing, numbers, and social skills. Overall, his medical history was inadequately documented, with noted gaps.

Blood tests were conducted to rule out platelet, coagulation, and endocrine disorders associated with this Proteus-like syndrome. Radiographic imaging studies assessed skeletal deformities, while abdominal ultrasonography yielded normal results. Magnetic resonance imaging (MRI) of the brain revealed no pathology. Biopsies were taken from the enlarged digit, nevus, and lipoma, and the child underwent surgery involving soft tissue debulking and physeal arrest to achieve a satisfactory functional and cosmetic outcome. Targeted analysis for pathogenic variants in *AKT1* [AKT Serine/Threonine Kinase 1; c.49G>A (p.Glu17Lys)], *PTEN* (Phosphatase and Tensin Homolog), and *PIK3CA* (Phosphatidylinositol-4,5-Bisphosphate 3-Kinase Catalytic Subunit Alpha) in the affected digit tissue returned negative results. Consequently, whole-exome sequencing (WES) of the same tissue was performed (Twist Exome Comprehensive on Illumina NovaSeq X). Given the child’s normal karyotype, we also performed comparative genomic hybridization using peripheral blood (SurePrint G3 Human Genome CGH+SNP Microarray Kit 4x180K, Agilent, Santa Clara, CA, USA).

## 3. Results

A 1.25 Mb copy number gain at 16p11.2-p11.1 [GRCh38/hg38 16p11.2(chr16:33836076 -35085876)x3], along with the deletion of the *GPRIN2* gene and two duplications in the region following *AKT1* at 14q32.33 (432 and 353 kb, respectively), has been reported. Corresponding studies within the family indicate that the 16p11.2 duplication was maternally inherited. WES analysis yielded results of limited significance concerning macrodactyly: one heterozygous pathogenic variant in *PRRT2* c.649delC (p.Arg217fs; located at 16p11.2) and one likely pathogenic splice variant in *SLC19A3* (c.1172+1G>A). The *PRRT2* c.649delC variant has been associated with benign familial infantile seizures and episodic kinesigenic dyskinesia, with or without infantile seizures, although there is growing evidence of incomplete penetrance [5,6,7]. Given the patient’s neurological status and the lack of sufficient documentation in his medical history, it was challenging to connect this mutation with his phenotype. Certainly, *PRRT2* mutations alone cannot explain macrodactyly; therefore, the WES findings were deemed inconclusive.

In this case, macrodactyly, lipomas, and nevi raised clinical suspicion for a Proteus-like syndrome or an overgrowth syndrome affecting the PI3K-PTEN-AKT1 signaling pathway. The two copy number variants (CNVs) near *AKT1* found in our patient were not likely to be pathogenic. On the other hand, the deletion of *GPRIN2* (G-Protein-Regulated Inducer Of Neurite Outgrowth 2) could be involved in our patient’s phenotype, as it has been reported as a candidate modifier gene in different syndromes, including 16p11.2-p12.2 duplication syndrome (a genomic condition differentiated from euchromatic variations in 16p11.2), which is associated with defects in digit formation [8,9]. Parental psychiatric disorders have been previously associated with inherited 16p11.2 duplications. In our case, the boy’s macrodactyly led to a diagnosis for both the mother and the son. Moreover, individuals with 16p11.2 duplication are expected to display more grossly dysmorphic features compared to deletion cases (including digit malformations), but there is no recognizable pattern except microcephaly (especially in mothers) [10]. However, no consistent pattern of physical abnormalities characterizes 16p11.2 duplications; signs and symptoms may vary even among affected members of the same family.

In summary, the genetic testing results for macrodactyly should trigger a multidisciplinary approach and detailed follow-up care. Further comprehensive studies are needed to define genotype–phenotype correlations. A diagnostic algorithm is essential in evaluating macrodactyly, particularly when syndromic or other pathological clinical features coexist.

## 4. Discussion

**Figure 2:**
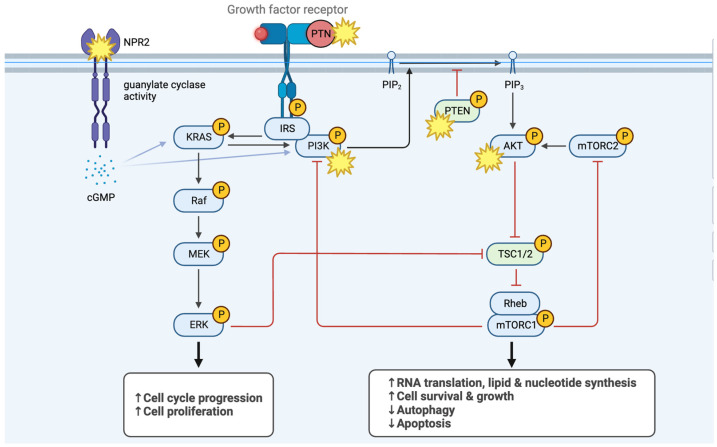
Simplified PI3K/AKT/mTOR and Ras/MAPK pathways and the associated mutation sites of macrodactyly.

**Figure 3:**
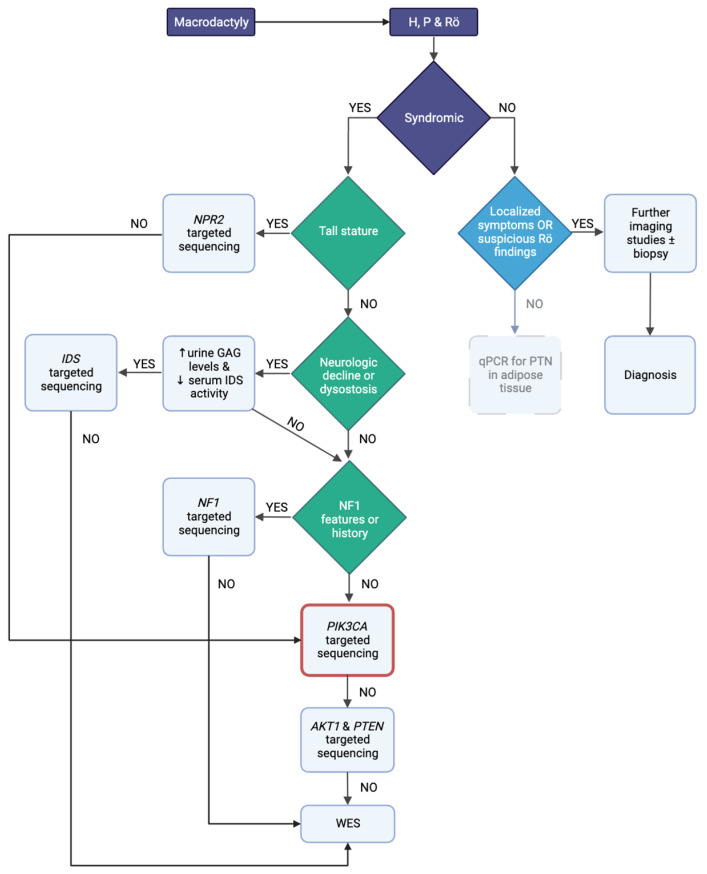
The proposed diagnostic algorithm for the investigation of macrodactyly. GAG = glycosaminoglycans; H = history; P = physical examination; Rö = radiography.

Macrodactyly is a rare condition, and surgical treatment is often insufficient unless the underlying pathology is unveiled. Most macrodactyly cases correspond to a post-zygotic somatic gain-of-function mutation in *PIK3CA* in varying proportions of cells within the affected digit. Figure 2 illustrates the PIK3K/AKT/mTOR and Ras pathways, and Table 1 lists all entities presenting with macrodactyly according to OMIM (Online Mendelian Inheritance in Man; https://www.omim.org/ (accessed on 17 February 2025) and relevant publications [11,12,13]. The established clinical diagnostic criteria for PIK3CA-related overgrowth spectrum (PROS) require the presence of a somatic *PIK3CA* mutation [11]. There are two significant setbacks when investigating macrodactyly: (a) while macrodactyly may appear isolated, it can be part of a syndrome prior to its onset; (b) it is questionable whether genetic testing can predict outcomes and disclose follow-up actions. After reviewing the literature, a diagnostic algorithm was built for these reasons (Figure 3).

Non-syndromic macrodactyly is more frequent than syndromic and multidigit involvement and is 2.6 times more prevalent than single-digit defects [3]. In non-syndromic cases, our primary concerns are to investigate whether macrodactyly is progressive or static and to decipher the traits of the affected tissue. Signs of inflammation, necrosis, and suspicious radiograph findings should trigger further investigations to eliminate underlying malignancies, hamartomas (including the Sturge–Weber sequence [12]), capillary malformations, compromised lymphatic system, and inflammatory processes. In the absence of localized symptoms and static cases, qPCR for pleiotrophin in adipose tissue can set the diagnosis, but this practice is not widespread [14]. Contrariwise, isolated macrodactyly is usually a congenital physical anomaly that can be recognized prenatally and may require genetic counseling and testing, although there is no such consensus or guideline [15]. Given the high prevalence of *PIK3CA* mutations in cases of isolated macrodactyly, WES is strongly recommended [1].

When syndromic features are present, and before proceeding with *PIK3CA* genetic testing, we must eliminate other less common diagnoses. Macrodactyly and epiphyseal dysplasia (especially of the halluces) have been associated with tall stature and pathogenic variants in the C-type natriuretic peptide gene and its receptors [16]. Mucopolysaccharidosis type II is a rare lysosomal storage disease that can present with macrodactyly. As it may sometimes have a mild clinical course, physicians should remain vigilant [17]. Historically, several reports have associated neurofibromatosis with macrodactyly (primarily affecting *NF1* and, even more rarely, *NF2*). Although there is no clear pathogenetic link, clinicians should eliminate this diagnosis [3,18]. Nevertheless, if genetic tests return negative for all the above instances, the investigations should always proceed with *PIK3CA*-targeted sequencing. Of interest, the consensus for the Testing Eligibility Criteria for Somatic *PIK3CA* Mutations notes that macrodactyly combined with genitourinary, gastrointestinal, and neurologic abnormalities (including autism and hypoglycemia) should prompt respective testing [11]. Currently, research employing targeted next-generation sequencing (NGS)-based methods to explore the genetic basis of macrodactyly holds much promise [19]. Targeted exome sequencing has recently become more accessible and has been added to clinicians’ armamentarium, while its data analysis seems to detect CNVs efficiently [1]. At this later stage, Sanger sequencing is inferior to targeted WES in terms of time and cost-effectiveness [20]. Based on the technical difficulties of genetic testing in these cases (mosaicism and challenges with tissue biopsy), negative WES should not be conclusive, and follow-up is warranted [21]. In general, molecular approaches in macrodactyly investigation can include gene-targeted testing (targeted analysis, single-gene testing, multigene panel) and comprehensive genomic testing (exome sequencing, genome sequencing), depending on the phenotype. Gene-targeted testing necessitates that the clinician identifies specific genes that may be implicated, whereas genomic testing does not [22]. The diagnostic yield of WES in neurodevelopmental disorders shows a clear benefit over CGH [23]. While not commonly used for overgrowth syndromes, evidence supports WES as a first-tier, cost-effective approach [24]. WES has also been used alongside RNA sequencing, but this approach is considerably too expensive [25].

It is essential to understand the phenotypes associated with PROS because, in many cases, they are associated with high-mortality risk, while surveillance and timely surgical and/or pharmacological therapy could significantly improve the quality of life in the affected patients. An international expert consensus statement on the standard-of-care clinical practice guidelines for individuals with PROS has recently been published [26].

Cancer incidence is not significantly increased among individuals with macrodactyly, apart from Wilms’ tumor, which is the only malignancy associated with PROS (2% of patients). Although there is insufficient evidence to demonstrate high risk, serial abdominal ultrasounds every 3–4 months until the age of 8 years are recommended in all children with a somatic *PIK3CA* mutation. One case of ovarian cystadenoma has also been reported with PROS [27]. *PIK3CA* mutations are prevalent in cancers that involve ectodermal or endodermal epithelia (e.g., endometrial and breast). Conversely, the affected tissue in PROS is either of mesodermal or neuroectodermal origin [28]. Although macrodactyly is a tumorous condition, it is not considered malignant, and no malignant transformation is anticipated [4]. However, studies of alpelisib, an alpha-specific PI3K inhibitor, in children with PROS have demonstrated efficacy and safety in alleviating symptoms [29]. Further, macrodactyly could be diagnosed with neurofibromatosis, tuberous sclerosis, and Proteus syndrome, all of which are strongly associated with neoplasms [12,13,18].

Surveillance measures in PROS include the measurement of growth parameters (including head circumference, length of arms, hands, legs, and feet) at each visit to the pediatrician, neurologic and psychomotor assessments, and a detailed examination of scoliosis and abdominal masses. Beyond the serial abdominal ultrasounds mentioned above, serial brain, spinal, and truncal MRI testing is indicated only in patients with pathologic findings in these respective regions. Consultation for endocrinopathies and hematological manifestations should be sought accordingly [26,30]. In our case, the success of the surgical treatment, the improvement of the patient’s psychomotor skills with therapy, the lack of new neurological or other pathological manifestations, and the genetic testing results led to follow-up with annual visits to the pediatrician. Nevertheless, the coexistence of macrodactyly with mild and nonspecific dysmorphic features, along with ASD and a history of psychomotor delay, prompted genetic testing in our case, making the prioritization of eliminating a PROS diagnosis essential. The clinical management of syndromic macrodactyly is well described by consensus guidelines. This report denotes that isolated macrodactyly also needs the attention of physicians and warrants a multidisciplinary approach as it can be, in fact, part of a syndrome. Genetic testing is not always conclusive regarding outcomes, and follow-up actions are complex to appoint.

## 5. Conclusions

Macrodactyly is a rare and challenging physical anomaly that usually reflects a somatic pathologic variant along the PI3K/AKT/mTOR pathway. Still, other diagnoses must be ruled out in the absence of a pathologic *PIK3CA* variant. In our case, macrodactyly-related investigations identified a 16p11.2 duplication, which is predominantly associated with this patient’s ASD and psychomotor delay but is not causally associated with digit malformation. Long-term results of macrodactyly surgical treatment vary significantly between centers, but tissue overgrowth seems to be recurrent, and secondary degenerative bone changes are expected. A multidisciplinary approach is required due to potential comorbidities and mental health issues. The advent of novel NGS tools has significantly transformed the diagnostic landscape for associated disorders, resulting in a marked reduction in the number of undiagnosed cases. However, there remain critical unmet needs, particularly in ensuring a smooth transition from pediatric to adult healthcare and establishing specialized centers for conducting clinical trials.

## Figures and Tables

**Table 1:** Phenotypes associated with macrodactyly.

Phenotype	Gene (Location)	Other Features
*PIK3CA*-related overgrowth syndrome (PROS)	*PIK3CA* (3q26.32)	Including (i) macrodactydy including fibroadipose overgrowth; (ii) CLOVE syndrome; (iii) CLAPO syndrome; (iv) epidermal nevus; (v) seborrheic or benign lichenoid keratosis; (vi) cerebral cavernous malformations 4; (vii) Cowden syndrome 5; (viii) MCAP; (ix) HHML; (x) muscle hemihypertrophy; (xi) facial-infiltrating lipomatosis; and (xii) specific cancers: breast, colorectal, gastric, hepatocellular, non-small-cell lung, and ovarian types.
Non-syndromic macrodactyly	*PTN* (7q33)	Pleiotrophin upregulation. PTN seems to activate PI3K signaling.
Klippel–Trenaunay–Weber syndrome	Diversity in its genetic landscape. Many cases display somatic mosaicism for a missense mutation in *PIK3CA*	Large cutaneous hemangiomata with hypertrophy of the related bones and soft tissues, venous varicosities, and half of patients with Kasabach–Merritt syndrome
Tuberous sclerosis complex (TSC)	*TSC1* (9q34.13) or *TSC2* (16p13.3)	Autosomal dominant. Cystic areas of bone rarefaction–especially for phalanges
Natriuretic Peptide Receptor 2 mutations	*NPR2* (9p13.3)	Including epiphyseal chondrodysplasia, Miura type. Macrodactyly is also seen with *NPR3* (Boudin–Mortier syndrome) and *NPPC* (alias *CNP*) variants.
*PTEN* hamartoma syndromes (with variable expression and age-related penetrance)	*PTEN* (10q23.31)	Including (i) Cowden syndrome 1: a hamartomatous disorder characterized by macrocephaly, facial trichilemmomas, acral keratoses, papillomatous papules, and an increased risk for the development of breast, thyroid, and endometrial carcinoma; (ii) Bannayan–Riley–Ruvalcaba syndrome: hamartomatous polyps of the gastrointestinal tract, mucocutaneous lesions, developmental delay, macrocephaly, lipomas, hemangiomas, and pigmented speckled macules of the glans penis in males, and an increased risk of developing neoplasms; (iii) Lhermitte–Duclos disease with dysplastic gangliocytoma of the cerebellum; and (iv) Proteus-like syndrome
Proteus syndrome	*AKT1* (14q32.33)	Partial gigantism of hands and feet, nevi, hemihyperthrophy, and macrocephaly; Elattoproteus syndrome and fibroadipose hyperplasia included
Mucopolysaccharidosis type II or Hunter syndrome	*IDS* region (Xq28)	X-linked recessive inheritance. Patients excrete excessive amounts of chondroitin sulfate B (dermatan sulfate) and heparitin sulfate (heparan sulfate) in the urine. Severe airway obstruction, skeletal deformities, cardiomyopathy, and neurologic decline.

CLAPO = capillary malformation of the lower lip, lymphatic malformation of face and neck, asymmetry of face and limbs, and partial/generalized overgrowth; CLOVE = congenital lipomatous overgrowth, vascular malformations, and epidermal nevi; HHML = hemihyperplasia multiple lipomatosis; IDS = iduronate 2-sulfatase; MCAP = megalencephaly-capillary malformation and polymicrogyria syndrome.

## Data Availability

The data presented in this study are available on request from the corresponding author due to privacy restrictions.
